# Ecological and genetic divergence between two lineages of Middle American túngara frogs *Physalaemus *(= *Engystomops*) *pustulosus*

**DOI:** 10.1186/1471-2148-10-146

**Published:** 2010-05-18

**Authors:** Heike Pröhl, Santiago R Ron, Michael J Ryan

**Affiliations:** 1 Institute of Zoology, University of Veterinary Medicine, Bünteweg 17, 30559 Hannover, Germany; 2 Museo de Zoología, Centro de Biodiversidad y Ambiente, Escuela de Biología, Pontificia Universidad Católica del Ecuador, Av. 12 de Octubre 1076 y Roca, Aptdo. 17-01-2184, Quito Ecuador; 3 Section of Integrative Biology, 1 University Station C09300, The University of Texas, TX78712, USA; 4 Smithsonian Tropical Research Institute, P.O. Box 0943-03092 Balboa Ancón, Republic of Panamá

## Abstract

**Background:**

Uncovering how populations of a species differ genetically and ecologically is important for understanding evolutionary processes. Here we combine population genetic methods (microsatellites) with phylogenetic information (mtDNA) to define genetic population clusters of the wide-spread Neotropical túngara frog (*Physalaemus pustulosus*). We measure gene flow and migration within and between population clusters and compare genetic diversity between population clusters. By applying ecological niche modeling we determine whether the two most divergent genetic groups of the túngara frog (1) inhabit different habitats, and (2) are separated geographically by unsuitable habitat across a gap in the distribution.

**Results:**

Most population structure is captured by dividing all sample localities into two allopatric genetic lineages. The Northern genetic lineage (NW Costa Rica) is genetically homogenous while the Southern lineage (SW Costa Rica and Panama) is sub-divided into three population clusters by both microsatellite and mtDNA analyses. Gene flow is higher within the Northern lineage than within the Southern lineage, perhaps due to increased landscape heterogeneity in the South. Niche modeling reveals differences in suitable habitat between the Northern and Southern lineages: the Northern lineage inhabits dry/pine-oak forests, while the Southern lineage is confined to tropical moist forests. Both lineages seem to have had little movement across the distribution gap, which persisted during the last glacial maximum. The lack of movement was more pronounced for the Southern lineage than for the Northern lineage.

**Conclusions:**

This study confirms the finding of previous studies that túngara frogs diverged into two allopatric genetic lineages north and south of the gap in the distribution in central Costa Rica several million years ago. The allopatric distribution is attributed to unsuitable habitat and probably other unknown ecological factors present across the distribution gap. Niche conservatism possibly contributes to preventing movements across the gap and gene flow between both groups. Genetic and ecological data indicate that there is the potential for ecological divergence in allopatry between lineages. In this context we discuss whether the Northern and Southern lineages should be recognized as separate species, and we conclude that further studies of pre- and post-zygotic isolation are needed for a final assessment. Identified population clusters should motivate future behavioral and ecological research regarding within-species biodiversity and speciation mechanisms.

## Background

A central goal of evolutionary ecology is to understand processes that determine the distribution of genetic diversity and population connectivity through gene flow. The current population genetic structure of a taxon is the result of many factors such as the dispersal capacity of the organism, the degree to which individuals of different populations recognize each other as potential mates, the connectivity of suitable habitats, historical events that impose geographical isolation (e.g. glaciations or elevation of mountains) and interactions with ecologically similar species or predators [1-4].

Isolation by distance [5] occurs when gene flow declines with increasing distance between pairs of populations, and is characteristic of the overall genetic population structure of many amphibians species [2,3,6-9]. Amphibians often exhibit strong site fidelity (philopatry, reviewed in [10]), have patchy geographical distributions due to specific and complex habitat requirements and low dispersal capacity [11]. Most studies of genetic population structure of amphibian show little or no genetic differentiation with moderate to high gene flow and dispersal rates at the local scale (<5-10 km), but stronger genetic differentiation at larger geographical scales (>15-20 km) [8,12,13]. Less common are pattern of panmixia among distant localities (*Bufo marinus*, [14]) or strong genetic differentiation across very small geographic distances (*Bufo calamita*, [9]).

Gene flow among populations is fundamentally related to genetic variability within populations. In areas where gene flow among populations is high, genetic diversity within populations is likely to be higher (but see [4]) than in areas with reduced gene flow (e.g. peripheral populations [15]). The most likely explanation for this phenomenon is reduced genetic drift and introduction of rare or new alleles in less isolated populations. Also, colonization events and bottlenecks are expected to have impacts on genetic diversity: generally older lineages are genetically more diverse than populations that have been founded by more recent colonization [2,16].

Multiple landscape features have recently been shown to be important for the magnitude of gene flow and thus for explaining population genetic structure in amphibians. Roads, mountain ridges and open or dry habitats apparently reduce gene flow between populations [7,8,17,18] while forests, wetlands and rivers are associated with increased gene flow [7,19]. For example, in two neotropical species of the genus *Craugastor*, mountains and dry forests present ecologically unsuitable habitat and act as barriers to gene flow resulting in diverged genetic lineages [18].

Both genetic and ecological divergence can lead to reproductive isolation and speciation. Ecological speciation is the evolution of reproductive isolation as a result of divergent natural selection in different environments and can proceed in the presence or absence of gene flow [20]. Several general scenarios for speciation have been identified including genetic differentiation, geographic distribution and shift in ecological niches [21]. These scenarios associate speciation processes occurring in sympatry, parapatry or allopatry with presence or absence of ecological divergence. A combination of environmental niche models with phylogeographic analyses offers the possibility to explore the role of geographic and ecological separation for speciation between diverging lineages.

Although phylogenetic and phylogeographic information has been increasing recently, few studies have been conducted simultaneously on both fine and large scale population genetics in tropical anurans [13,22,23]. In the Neotropics, phylo-geographic studies have been mainly limited to craugastorid [13,18], strabomantid [24,25] and dendrobatid frogs [26-30]. These studies often revealed genetic lineages within species [23,26,27,30], rectified the taxonomic relationship between species or species groups [28], or uncovered previously unknown species diversity [24]. Examining ecological divergence in a phylogenetic context has been applied to only a very limited number of Neotropical frogs. These studies demonstrated climatic specialization along temperature and seasonality axes and identified important speciation mechanism for some clades [21,31]. For most Neotropical frogs, however, there is no information about their genetic population structure, genetic diversity within the species, presence of genetic lineages, or even species status and variability in ecological requirements. Still less is known about the relationship between genetic divergence, reproductive isolation and ecological divergence, and the relative importance of all these aspects for speciation.

To our knowledge the spatial genetic organization [32-35] and reproductive behavior (review in [36,37]) of the túngara frog *Physalaemus *(= *Engystomops*) *pustulosus *is more thoroughly documented than any other Neotropical frog species. The distribution of the túngara frog ranges from northern Mexico to the Caribbean coast of northern South America where these frogs are abundant in dry and wet lowland forests. Allozymes analysis by Ryan et al. (1996) [32] found two genetic lineages of túngara frogs: a Northern lineage containing populations from Mexico to northern Costa Rica and a Southern lineage ranging from western Panama to northern South America (Venezuela, Columbia, Trinidad). This was confirmed by Weigt et al. (2005) [35] who added an analysis of mtDNA (COI) to the previous data set. The time of the separation between both lineages (~6-9 Myr, [35]) was estimated to have occurred prior to the final closing of the Panamanian land bridge (3.1-2.8 Myr, [38]). These results were further confirmed by a fine-scaled molecular study in an area of Costa Rica and Panama that encompassed both genetic lineages [34] and a phylogenetic study of the *P. pustulosus *species group [39]. The Northern and Southern lineages are separated by a distribution gap of about 200 km in Central Costa Rica [40].

The aim of this study was threefold: (1) to analyze the potential for ecological divergence between the Northern and Southern lineages, (2) to test the persistence of the geographic barrier between the Northern and Southern lineages during the last glaciation and explore the hypothesis of allopatric divergence, and (3) to provide a more detailed population genetic analysis across 25 localities for the aforementioned area (Figure 1 in [34]) and compare the population genetic structure to that of other amphibian species.

**Figure 1:**
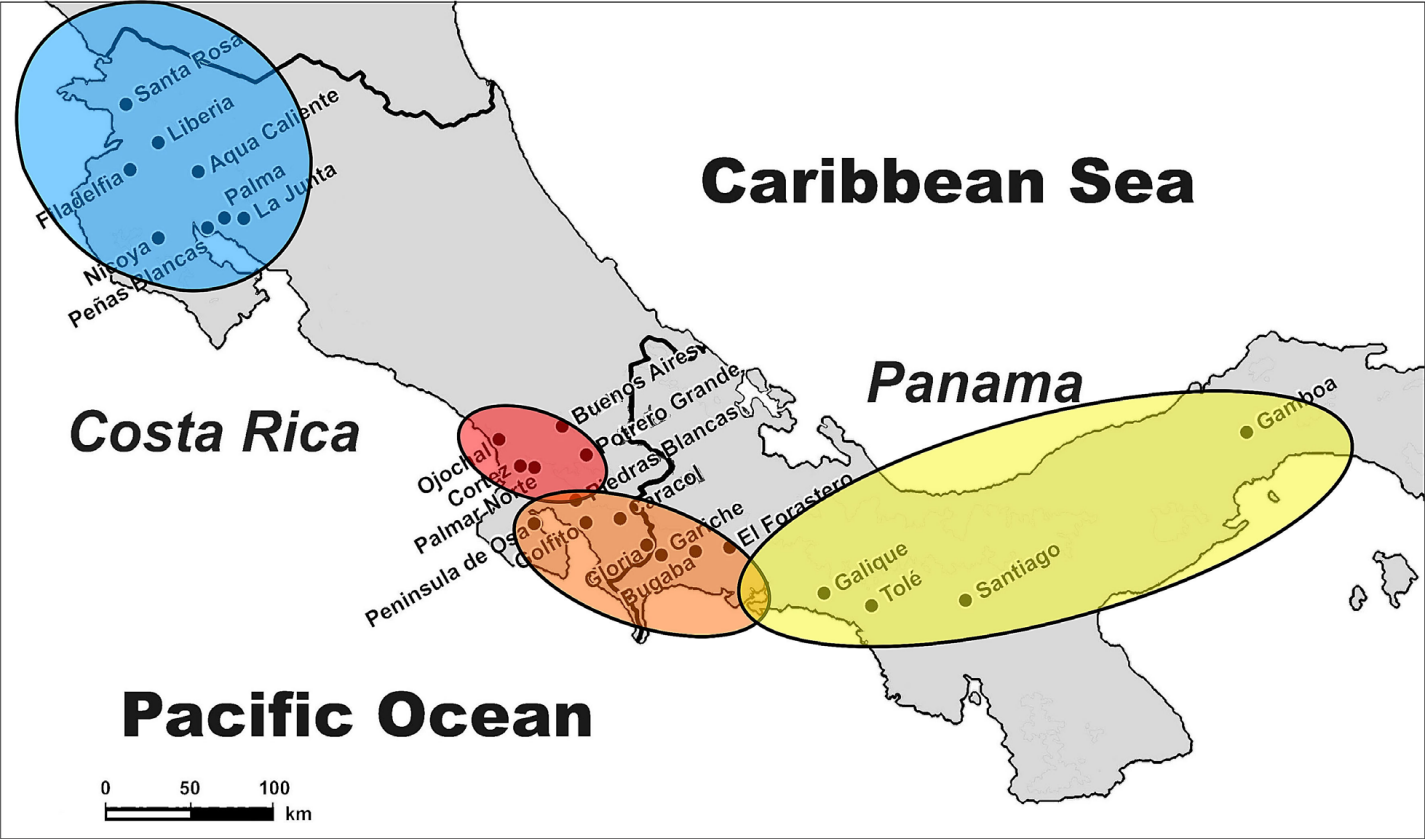
**Map of study area**. Map with sampling localities with genetic clusters identified by Structure (Figure 2, this study). Blue = North, Red = South_1, Orange = South_2, Yellow = South 3. The figure was modified from Figure 1 published in Ref. [34].

We first conduct a Bayesian analysis to detect population subdivision and zones of admixture based on highly polymorphic nuclear markers. We further calculate population differentiation (F_ST _and R_ST_) between inferred populations and assign individuals to their most probable population of origin for estimating migration between populations. Our analyses include calculation of genetic diversity across the investigated area. We also estimate divergence time between the Northern and Southern lineage and between population clusters based on sequence divergence in the mitochondrial *Cyt B *gene. Then we integrate our parameter estimates obtained from classical population differentiation analyses (F-Statistics) and recently developed Bayesian statistics. Thus we link classical measures of population structure (F_ST _& R_ST_) to population structure inferred from the clustering method, migration patterns deduced from assignment tests and divergence time estimates. Furthermore we conduct environmental niche modeling based on climatic and altitude parameters to estimate the potential for ecological niche divergence between genetic lineages, to measure ecological landscape connectivity across the distribution gap and to predict the distribution of past and current suitable habitat. The allopatric distribution of the Northern and the Southern lineage suggests that genetic divergence is a result of geographic isolation. This evidence, however, is insufficient to support an allopatric model of divergence because distribution ranges could have been in contact in the past. To explore this possibility, we project niche models to the last glacial maximum. A result showing equal or higher habitat unsuitability across the distribution gap during the last glacial would provide further support for the scenario of allopatric divergence. We discuss the possibility that speciation has occurred between the Northern and Southern lineage based on the combined evidence of genetic and ecological data together with previously collected behavioral data.

Moreover, this thorough population genetic and ecological analysis on a species for which the communication system, mating system and mate choice strategies are well studied will offer the opportunity to detect important areas (such as hybrid zones, areas of low or high genetic diversity and areas that differ ecologically) for studying new aspects of behavior in relation to the ecology and genetic composition of the population.

## Results

### Microsatellites

**Figure 2:**
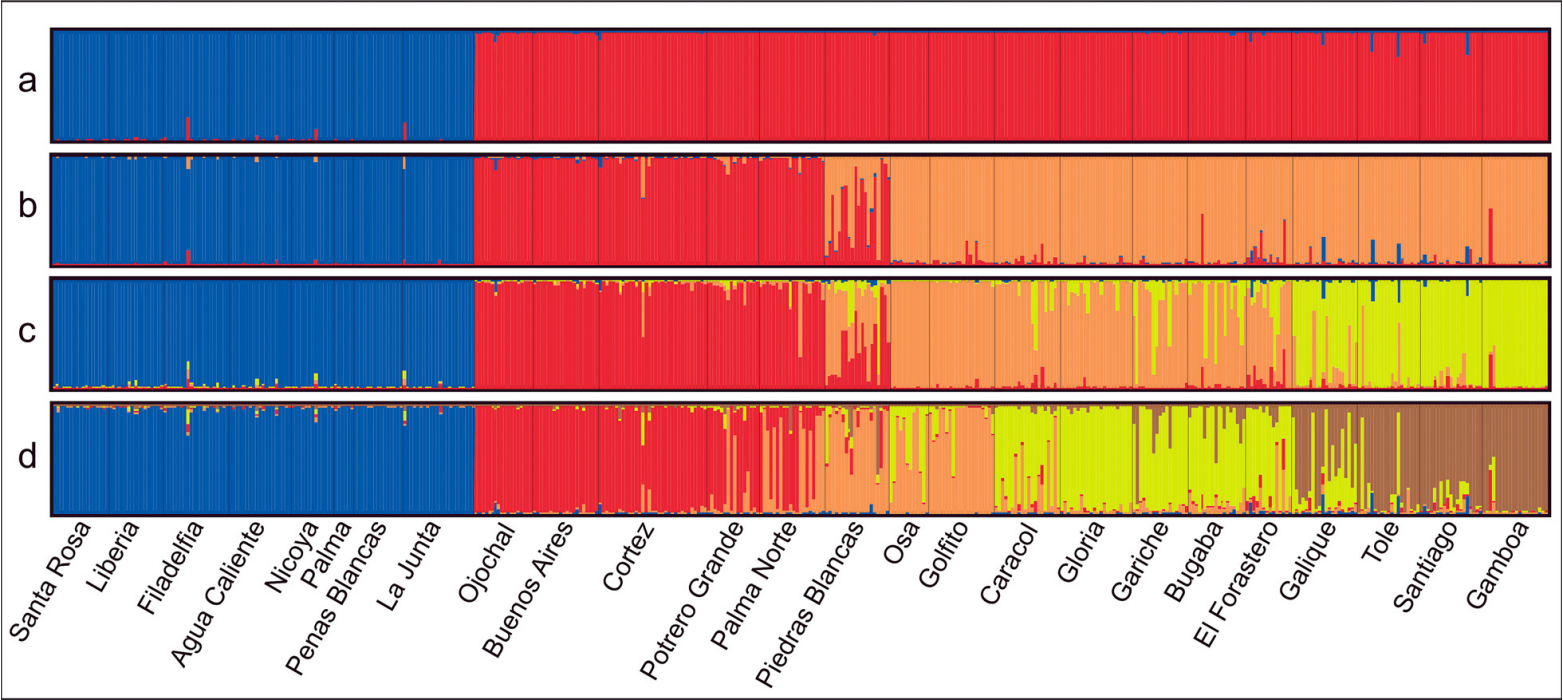
**Population structure of Middle American túngara frogs**. Results of the Bayesian analysis for identifying population structure for K = 2, K = 3, K = 4 and K = 5. The first and most significant separation is between the Northern and Southern lineage (K = 2). However the Southern lineage can be further subdivided in several genetically distinct population clusters.

**Figure 3:**
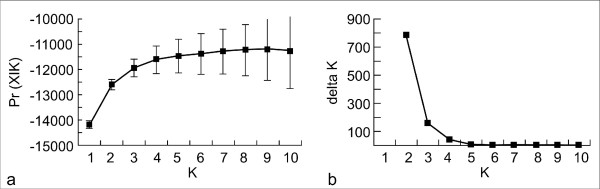
**Posterior probability for population cluster**. a) The log likelihood [Pr(X I K)] for a given number of population clusters (K) in Middle American túngara frogs. The graph shows that most of the population structure is captured by setting K = 3 or K = 4. For higher Ks the likelihood is not or only very slightly increasing. b) ΔK in relation to the number of clusters (K) [59]. The graph shows that ΔK is highest for K = 2, which relates to the geographic division of túngara frogs by the gap in a Northern and Southern lineage. However there is still a relevant change in the Pr(X I K) by increasing K to 3 and 4, which relates to a further subdivision of the Southern lineage in several population clusters.

Population structure: Most population structure was captured by dividing all sample localities in two genetic lineages (Figure 1, Figure 2a) The southern lineage, however, was further subdivided in two or three sub-clusters (Figure 2b, c), while the Northern lineage remained undivided even by increasing K to 10. We decided that the most probable population structure is given for K = 4. We base our decision on the following arguments: The likelihood of the data increased considerable when raising K = 2 to K = 3 and K = 4 (Figure 3a). Afterwards Pr (X|K) increased only insignificantly. On the other hand, there was still a substantial ΔK for K = 4, but for higher K values ΔK remained close to zero (Figure 3b). Also, the assignment of individuals to a population cluster became less clear for K = 5 (Figure 2d), and this was increasingly the case for higher values of K (data not shown). Conducting the analysis separately for the Northern and Southern lineage resulted in the same geographic-genetic pattern, i.e. by analyzing the Southern lineage alone ΔK_south _was highest for K_south _= 3. In the following we name the identified population clusters North (blue), South_1 (red), South_2 (orange) and South_3 (yellow areas; Figure 1, Figure 2c).

The Northern lineage (or cluster) was located in North Costa Rica, i.e. in the north of the gap. Within the Southern lineage Structure found an admixture zone (South_Admix, red-orange) in Piedras Blancas which was evident for K = 3 and K = 4 (Figure 2b, c). All individuals in this population possessed genetic material from the two (for K = 3) or three (for K = 4) southern clusters. The cluster adjacent in the north-west of the admixture zone ranged from the southern edge of the gap (Ojochal) through the lowland areas along the coast and the lowland areas in the *Valle de General *(Buenos Aires) and *Valle de Cotobrus *(Potrero Grande). The cluster adjacent towards the south of the admixture zone ranged until El Forastero in Western Panama. The sample locality on the Peninsula Osa was included in this cluster. Sixty kilometers to the east, the third sub-cluster of the southern genetic group ranged from Galique to Gamboa, the eastern most locality (Figure 1).

In order to determine the degree of genetic differentiation among clusters we calculated F_ST _und R_ST _values between each pair of clusters and the admixture zone in Piedras Blancas (Table 1). All pairs of clusters were significantly different in estimates of both F_ST _or R_ST_. As expected, the Northern lineage was most diverged from all other clusters. Comparing the northern cluster with the three southern clusters it is notable that the F_ST _was highest between North and South_1 (the geographically closest cluster) while the highest R_ST _values were found between North and the admixture zone. R_ST _values between the admixture zone and both adjacent clusters were non-significant. The proportion of membership inferred by Structure for each sample locality to the four clusters was for Piedras Blancas 37% to South_1, 48% to South_2, 13% to South_3 and 2% to North. This was not in full accordance with the F_ST _and R_ST _values: F_ST _values between Piedras Blancas and South_2 or South_3 were nearly equal and the R_ST _value was smallest for South_1. For pairwise R_ST _values see [Additional file 1].

**Table 1:** F and R-Statistics

	North	South_1	South_Admix	South_2	South_3
North	-------------	0.7173	0.8580	0.7810	0.7841
South_1	0.2246	--------------	0.0021*	0.0300	0.0758
South_Admix	0.2145	0.0814	----------------	0.0043^○^	0.0268^∇^
South_2	0.2109	0.1234	0.0441	--------------	0.0854
South_3	0.1893	0.1154	0.0437	0.0530	-------------

F_ST _(below) and R_ST _(above diagonal) values between population clusters previously identified by Structure. For each comparison P < 0.0001, except *P = 0.25, ^○^P = 0.29, and ^∇^P = 0.041

Gene flow and migration: In the population cluster North between 17% and 77% of individuals reached the highest assignment score for their home population (mean = 48%). 52% of individuals were assigned to another sample locality within the same population cluster (range 23-83% for each population) [Additional file 2]. In general, in the southern clusters a higher percentage of individuals was assigned to their home population: In South_1 60-95% (mean 75.4%) of individuals, in South_2 41-100% of individuals (mean 73.9%), in South_2 58-90% of individuals (mean 75.5%) per population [Additional file 2]. Only in the cluster South_3 for several individuals (range 10-26% per population, mean = 16.75%) the highest assignment score was for a sample locality in another population cluster (South_2: 11 individuals; South_1: 2 individuals). Overall, the results point to higher gene flow among populations in the cluster North than in the southern clusters. In the admixture zone (Piedras Blancas) the highest score in 17 individuals was for the home population, in 2 individuals for population Palmar Norte (South_1), and in one individual for Bugaba (South_2). Classical migration rates based on F_ST _and R_ST _values were small between North and the southern clusters and much higher among the southern clusters (Table 2).

**Table 2:** Migration rates among population clusters

0	North	South_1	South_Admix	South_2	South_3
North	-------------	0.1001	0.0413	0.0701	0.0688
South_1	0.8660	--------------	118.79	8.0833	3.0394
South_Admix	0.9182	2.8364	----------------	57.889	9.3654
South_2	0.8915	1.7825	5.4318	--------------	2.6911
South_3	1.0727	1.9239	5.4318	4.4669	-------------

Migration rates per generation N_m _[5] among population clusters previously identified by Structure, based on F_ST _(below) and R_ST _(above diagonal) values.

At the sample locality level the software GENECLASS2 detected 24 individuals for which the probability that the individual is a resident was <0.01 [Additional file 3]. These individuals are assumed first generation migrants. No migrants were detected between the Northern and Southern lineages. Most migrants (N = 6) were found in the northern cluster. Here several individuals from Liberia (N = 4) seem to have migrated to other sample localities. Within the cluster South_1 five individuals seemed to have migrated between populations. For South_2 this was true for four individuals. In the cluster South_3 five individuals were recognized as migrants from very distant source populations either in South_1 or South_2.

Genetic diversity: Mean allelic richness was higher in the population clusters south of the gap than in the cluster north of the gap (North: mean = 4.287; South_1: mean = 5.526; South_2: mean = 5.530; South_3: mean = 6.274) [Additional file 4]. Allelic richness differed significantly among population clusters (permutation test: two-sided P < 0.001). In general, allelic richness per population or cluster increased from the northern most localities towards South America. One fact that needs to be emphasized is that populations located somewhat aside from the main route along the Pan American Highway [Filadelfia (3), Nicoya (5), Peñas Blancas (7), Buenos Aires (10), Osa (15), Golfito (16)], possessed lower allelic richness than their neighbor populations. Also the two populations flanking the gap [La Junta (8) and Ojochal (9)] retained lower allelic richness.

**Figure 4:**
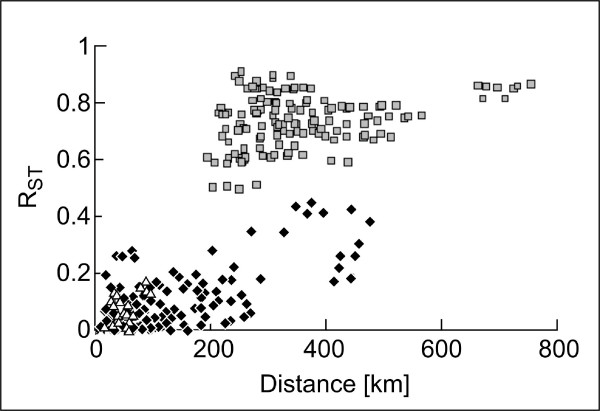
**Isolation by distance**. Genetic distance (R_ST _values) versus geographic distance (in km) as population pairwise comparisons in Middle American túngara frogs. Grey squares represent distances between one population from the North and one population from the South; White triangles represent distances between two populations from the North, and black diamonds represent distances between two populations from the South.

Isolation by distance: For the total range, genetic distance (R_ST_) and geographic distance were highly correlated (r = 0.79, P < 0.000001). 62% of the variation in genetic differences were explained by geography (R^2 ^= 0.62). For the Northern and Southern lineages separately the correlation coefficients were lower but still significant (North: r = 0.59, P < 0.0053, R^2 ^= 0.34; South: r = 0.66, P < 0.0036, R^2 ^= 0.44). Figure 4 shows the correlation between geographic and genetic distance within the Northern and Southern lineage. Inspection of the graph also reveals the effect of geographic and temporal separation between the northern and southern túngara frogs: genetic distances between pairs of localities from both the Northern and the Southern lineages were considerable higher in comparison with localities within the same lineage separated by a similar geographic distance.

### Cytochrome B

Genetic distance and time of divergence: The mean p distance (uncorrected nucleotide difference) between the Northern and Southern lineage was 0.074 ± 0.001 (overall mean ± SE = 0.045 ± 0.005), while the TN distance was calculated to be 0.081 ± 0.012 (overall: 0.049 ± 0.006). Distances (uncorrected p distances, TN distances) between genetic clusters are presented in Table 3. Whereas all distances between the Northern and the Southern lineages were between 0.065 and 0.087, the distances among groups of the Southern lineage were substantially lower. Interestingly the distance of the Northern lineage was lowest to South_2. Estimated divergence times between North and South were between 4.6 to 5.6 Myrs (Table 3, comparison between population clusters), while southern clusters diverged ~1.4 to 3.9 Myrs ago. Within-cluster divergence was lowest for North and increased towards the south (Table 4).

**Table 3:** Evolutionary distances between population clusters

	North	South_1	South_Admix	South_2	South_3
North	------	0.078/0.086	0.071/0.077	0.065/0.071	0.079/0.087
South_1	5.57	--------	0.011/0.011	0.020/0.021	0.054/0.057
South_Admix	5.07	0.071	--------	0.013/0.014	0.052/0.054
South_2	4.64	1.43	0.928	--------	0.047/0.049
South_3	5.64	3.86	0.037	3.36	----------

Uncorrected p distances/TN distances (above) and estimated divergence times (below) based on uncorrected p distances in MYR assuming a pair-wise mutation rate of 1.4% per Myr.

**Table 4:** Evolutionary distances within population clusters

	North	South_1	South_Admix	South_2	South_3
R_ST_	0.060 ± 0.054	0.019 ± 0.054	----	0.078 ± 0.085	0.167 ± 0.107
TN	0.000 ± 0.000	0.004 ± 0.001	0.019 ± 0.006	0.011 ± 0.003	0.027 ± 0.005

R_ST _(mean ± SE, based on microsatellites) and TN distances (based on *Cyt B *sequences) between sample localities within population clusters.

**Figure 5:**
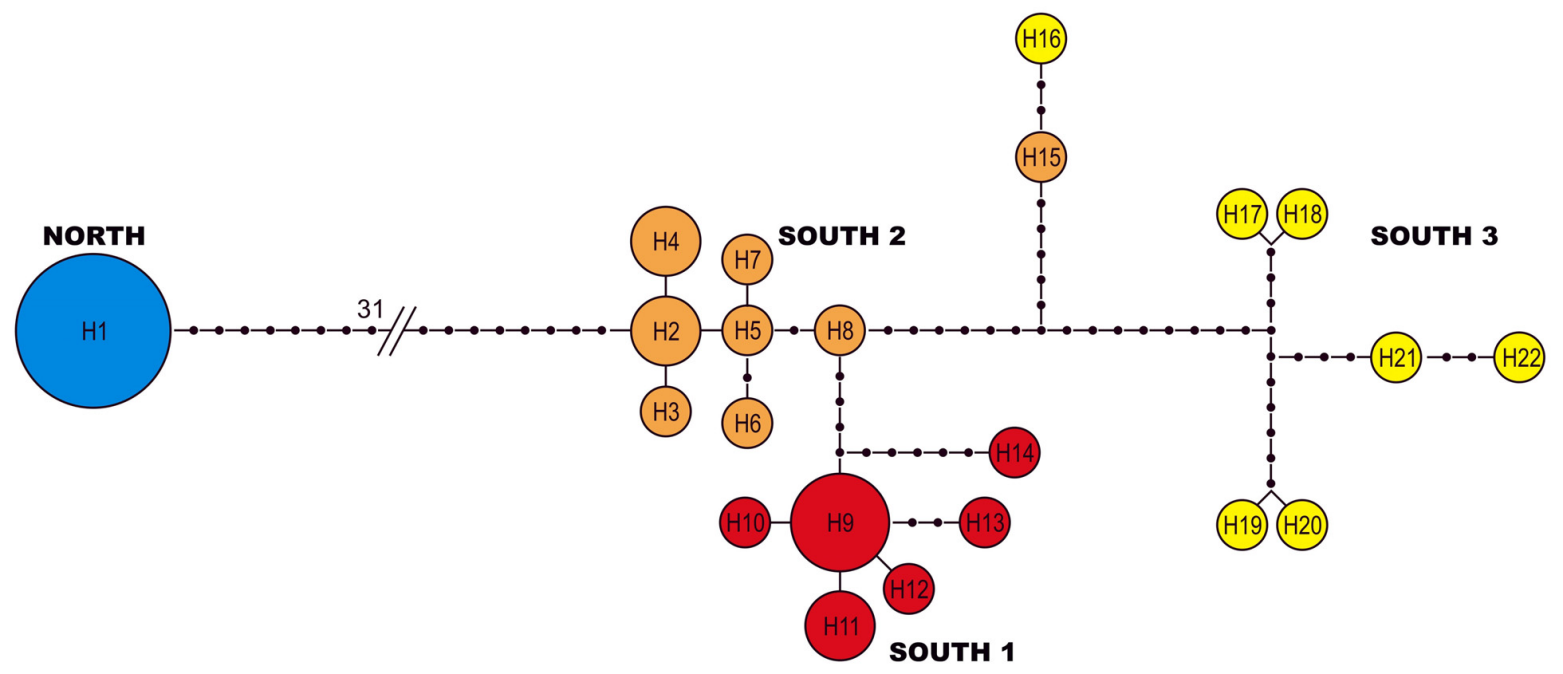
**Haplotype network**. Haplotype network of *Cyt B *sequences for Middle American túngara frogs. Blue haplotypes have been found in the northern genetic group (North), red, orange and yellow haplotypes belong to South_1, South_2 and South_3 respectively. One sequence of haplotype 4 (orange) and one sequence of haplotype 9 (red) are from Piedras Blancas, the admixture zone in South Costa Rica as identified by Structure.

The haplotype network (Figure 5) reveals three main genetic clades, one with two subclades, which correspond nearly perfectly with the population cluster detected by the Bayesian analysis based on microsatellites: 1. North (haplotype 1), 2. South_1 (haplotypes 9-14) and South_2 (haplotypes 2-8) as two subclades, and 3. South_3 (haplotypes 17-22). The two sequences from Piedras Blancas (Admixture zone) fall into South_1 (haplotype 9) and South_2 (haplotype 4). One sequence from the group South_2 (El Forastero 2, haplotype 15) clustered together with one sequence from South_3 (Galique 1, haplotype 16). The geographical divide between these two clusters was somewhere between El Forastero and Galique which are ~60 km apart (Figure 2c). The haplotype network confirms the closer relationship between North and South_2 than between North and South_1.

Diversity indices: Molecular diversity as measured by number of haplotypes (relative to number of individual sequences), polymorphic sites and nucleotide diversity was lowest in the Northern lineage (Table 5). In fact, only one haplotype was found in the North. All diversity indices increased from North towards the southern populations and were highest in population cluster South_3.

**Table 5:** *Cyt B *diversities at different levels of population structure

	N	h	s	Hd	π
All	37	21	73	0.913 ± 0.035	0.0452 ± 0.00315
					
North	10	1	0	0.000 ± 0.000	0.0000 ± 0.0000
South	27	20	57	0.963 ± 0.025	0.0291 ± 0.0037
					
South_1	9	5	7	0.806 ± 0.120	0.0036 ± 0.0013
Admix	2	2	9	1.000 ± 0.500	0.0185 ± 0.0093
South_2	9	8	21	0.972 ± 0.064	0.0101 ± 0.0047
South_3	7	7	35	1.000 ± 0.076	0.0263 ± 0.0055

*Cyt B *diversities over all populations, the Northern and Southern lineage and population clusters. Number of individual sequenced (N), number of haplotypes (h), number of polymorphic sites (s), haplotype diversity (Hd, mean ± SD) and nucleotide diversity (π, mean ± SD).

### Ecological Niches

**Figure 6:**
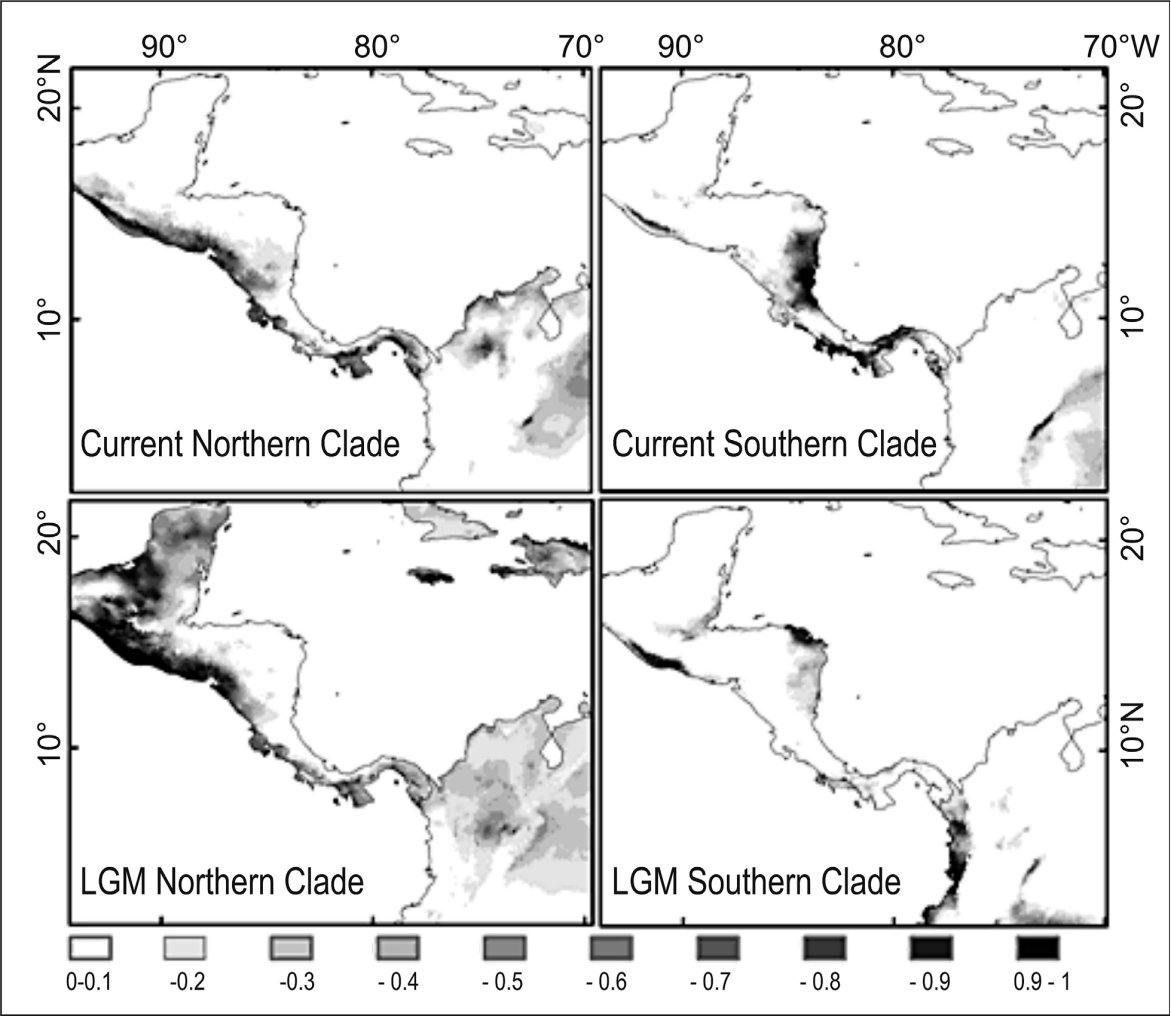
**Niche modeling**. Results for ecological niche modeling depicting predicted habitat suitability for túngara frogs in Middle America. Higher values indicate higher habitat suitability. "LGM" models are predictions for the Last Glacial Maximum (~21,000 years ago) based on paleoclimate models. See text for details.

The niche models generated with Maxent predicted the occurrence of suitable habitat better than random models (*P *< 0.001 in all 100 tests for the Southern lineage; *P *< 0.001 in all 10 tests for the Northern lineage). High model performance was also demonstrated by high values of the area under the curve (AUC) for the ROC analysis (AUC ≥ 0.981 for both models) indicating better than random predictions (0.5 = random, 1 = maximum). The models generated by Maxent (logistic output) consisted of maps with logistic values (LV) ranging from 0 (unsuitable habitat) to 1 (maximum suitability). The model for the Northern lineage predicted regions with high suitability in the dry forests and pine-oak forests of the Pacific basin from northern Costa Rica to southern Mexico (Figure 6). The model for the Southern lineage predicted regions with high suitability in the Pacific moist forests of Provincia Puntarenas (Costa Rica) and western Panama. High suitability was also present in the Atlantic moist forest of central Panama (Figure 6). Suitable habitat of the Northern lineage showed a lower annual precipitation than for the Southern lineage (North: mean ± SD = 1941.9 ± 409.4 mm; South: mean ± SD = 3078.7 ± 778.8 mm); also the mean annual temperate was on average colder in the North than in the South (North: mean ± SD = 25.52 ± 1.94°C; South: mean ± SD = 26.0 ± 0.84°C). There were significant differences in precipitation between the localities for the Northern lineage and the Southern lineage (Student's *t *= 5.21, *P *< 0.001). Differences were not significant for mean annual temperature (*t *= 0.681, *P *= 0.512). The paleoclimate models suggested significant changes in the location of suitable habitat with opposing trends in range size between both genetic groups (Figure 6). Under a logistic value (LV) threshold of 0.1 for presence-absence, the suitable range for the Southern lineage in Middle America, east from the gap, increased from 26,433 (last glacial maximum) to 66,569 km^2 ^(current). For the Northern lineage, the suitable range in Middle America, west from the gap, decreased from 1,234,558 km^2 ^(last glacial maximum) to 421,603 km^2 ^(current).

Connectivity and suitability across the distribution gap in Costa Rica: The analyses of suitability and connectivity suggested that the distribution gap in Costa Rica persisted during the last glacial maximum. Suitability for the Northern lineage across the gap was not significantly different between the present and the last glacial maximum (Mean LV current = 0.496, SD = 0.228; Mean LV last glacial = 0.489, SD = 0.195; paired *t *= 0.868, *P *= 0.387). For the Southern lineage, however, conditions deteriorated significantly (Mean LV current = 0.278, SD = 0.279; Mean LV last glacial = 0.052, SD = 0.086; paired *t *= 8.659, *P *< 0.001). The analysis of habitat connectivity suggested similar trends. For the Northern lineage, habitat resistance to reach the southern range was almost equal for current conditions and the last glacial maximum (least-cost distance path was 6.79 and 6.78, respectively). For the Southern lineage, resistance was much higher during the last glacial (14.94) than under current conditions (8.08). Taken together, these results suggest that geographic separation between genetic lineages persisted during glacial events.

## Discussion

Our analyses show that the two genetic lineages of túngara frogs in Middle America differ genetically and reside in ecologically different habitats. The Northern lineage is genetically homogenous while the Southern lineage consists of several population clusters: one in South Costa Rica close to the distribution gap between the lineages (South_1), one ranging from South Costa Rica to Western Panama (South_2), and the last one ranging from Western Panama towards East Panama (South_3). One sample locality in South Costa Rica (Piedras Blancas) was identified as a contact or admixture zone between South_1 and South_2. In general, the genetic pattern revealed by microsatellites is confirmed by sequences of the mitochondrial gene *Cyt B*. Genetic diversity is lowest in the Northern lineage and increased from South Costa Rica towards East Panama. In contrast, gene flow between sample localities is higher in northern Costa Rican than in the southern population clusters. Isolation by distance explains a large amount of the genetic variation in both lineages; however, time of divergence between population clusters also plays a significant role for genetic differentiation.

Environmental niche modeling revealed that the Northern and the Southern lineage differ significantly in habitat type. Suitable habitat for the Northern lineage is drier and occurs in pine-oak forests, while the Southern lineage is associated with moist forests. Annual precipitation is significantly higher for the habitat of the Southern lineage. Since the last glacial maximum suitable habitat decreased for the Northern lineage but increased for the Southern lineage. The habitat connectivity across the distribution gap in central Costa Rica was lower during the last glacial maximum, suggesting that ecological factors have prevented secondary contact from at least the Pleistocene to the present. This result is consistent with the large divergence time and the absence of migration between lineages recovered by two independent analyses (see below).

### Comparison of microsatellite and *Cyt B *analyses

Despite two different types of molecular markers (nuclear and mitochondrial) and different statistical methods (Bayesian cluster analysis versus haplotype network), both molecular approaches resulted in the same genetic population structure of Costa Rican and Panamanian túngara frogs. This indicates that the revealed population structure is fairly static given that mtDNA indicates ancient, historical events while microsatellites indicate more recent processes. Moreover, the different genetic distance measurements between population clusters (excluding the zone of admixture) are highly correlated (F_ST_-R_ST_: r = 0.89 P = 0.02; F_ST_-uncorrected p distance: r = 0.69, P = 0.13; R_ST_-uncorrected p distance: r = 0.88, P = 0.02, N = 6).

### Pattern of population structure in amphibians and isolation by distance

In general, amphibian populations display a high level of spatial genetic structure, mainly when interpopulation distances exceed several kilometers [12]. Túngara frogs also show this pattern as populations are genetically distinguishable at a very local level. Lampert et al. (2003) [33] found significant F_ST _values (>0.014) between populations that were on average three to four km apart (see Figure 2, [33]), a similar pattern was found in comparing island populations [41]. All studies, including this one, found a significant effect of isolation by distance, i.e. local migration takes place between neighboring populations, but over longer distances gene flow decreases. Our Bayesian Structure analysis did not further subdivide the Northern lineage into subpopulations and the assignment and migration analyses points to high levels of gene flow between sample localities. Pairwise R_ST _values ranged from 0.00 to 0.17 in the Northern lineage and 11 of the 28 population comparisons are significant, i.e. although R_ST _values ≥ 0.05 - 0.17 indicate significant genetic differences Structure still assigns them to the same genetic cluster. Our results suggest that genetic drift causes genetic differentiation while subpopulations are genetically connected by migrating animals.

Marsh et al. (2000) [42] showed that túngara frogs have a breeding neighborhood of less than 10 m; that is, at smaller distances males tend to move among breeding sites but not so at distances of 10 m or more. On the other hand, Marsh et al. (1999) [43] showed that these frogs did routinely move among ponds of distances of 200 m. This pair of studies suggests that túngara frogs are relatively philopatric but do possess significant dispersal abilities. Nevertheless, we seriously doubt that an individual túngara frog is able to cross distances of 20 km or more without the aid of human transport as the analysis of first migrant detection might suggest. Instead, we suggest that túngara frog subpopulations are well connected through ponds and puddles available for breeding during the rainy season as suggested by continuous suitable habitat between Liberia and Santa Rosa (Figure 6). As the túngara frog is well known to breed in areas of human disturbance, habitat disruption might not hinder and might even facilitate dispersion rather than impede it. Since the distance between our sample localities is on average 30 km, the apparent gene flow at such distances is probably established by substantial movements among breeding ponds that are much closer to each other. Our data indicate that the túngara frog is ecologically similar to the natterjack toad (*Bufo calamita*) that is known as a pioneering species with a high dispersal capacity, quickly colonizes early-successional habitats and displays low population structure at the local level, increasing population structure at larger distances and exhibits significant isolation by distance [9].

In the North, the assignment of individuals to their population was lower than it was in southern populations [Additional file 2], where more individuals were assigned to their true locality. This indicates either higher gene flow in the North than among southern populations, or a population bottleneck followed by population expansion. The habitat topography of South Costa Rica is more complex than North Costa Rica. In North Costa Rica no mountains separate different sample localities. In South Costa Rica, Buenos Aires (10) and Potrero Grande (12) are isolated from other sample localities by the *Fila Costeña *mountain range which parallels the coast. Nevertheless, gene flow between Palmar Norte, Cortez and Potrero Grande seems to be high while Buenos Aires appears to be more isolated. It is possible that gene flow between these three sample localities is conveyed by rivers. The *Rio Coto Brus *passes Potrero Grande, downriver it joins the river *Rio General *to form the *Rio Terraba *which connects to Palmar Norte and Cortez. Also, Osa (15) and Golfito (16) seem to be genetically more isolated within the southern populations then are the rest of the southern populations. Osa is more remote from other sample localities on the Peninsula Osa, while Golfito is located close to the coast and is separated from the lowland areas with the other sample localities by smaller mountains. Genetic assignment to the other sample localities is also lower in populations close to the distribution gap between La Junta (8) and Ojochal (9) indicating that gene flow and genetic diversity are reduced in peripheral populations. There is gene flow across the admixture zone of Piedras Blancas, especially towards South_1, but the high assignment to the home population indicates that gene flow is reduced as compared to genetically more homogenous areas. Another lowland area of high gene flow is between Gloria (18), Gariché (19) and Bugaba (20). Six individuals found in Gloria were genetically assigned with the highest probability to Gariché and eight individuals from Gariché were assigned with the highest probability to Gloria. In the third southern population cluster some individuals are assigned to South_2 [Additional file 2] and some "first generation migrants" stem from very distant home populations [Additional file 3: in Gamboa: 2 individuals have been assigned to Cortez and Caracol, ≥400 km distant] an observation that suggests ancient and current migration between both clusters.

### Divergences times

Our data are consistent with the proposition that túngara frogs invaded Middle America from South America at least twice [35]. The calculation of divergence times based on COI and a mitochondrial mutation rate of 0.69% per Myr between northern and southern túngara frogs in the study of Weigt et al. (2005) [35] resulted in an estimated divergence time of 6.35 MYA (5.04-7.66) while the parametric Bayesian MCMC method calibrated by the rise of the Ecuadorian Andes estimated the time of divergence as 8.61 MYA (95% confidence intervals: 4.07-13.3). Our calculations resulted in a divergence time of approximately 5 Myr between the Northern and Southern lineage which falls into the lower end of the confidence intervals calculated by Weigt et al. (2005). The difference between both studies in average divergence time estimates very likely rests upon the different geographical dimensions, i.e. in this study sampled populations next to the gap are geographically closer to each other (232 km versus 384 km [32,34]) and the sampled area covers a smaller geographic range.

### Ecological niches

Besides being genetically diverged, the Northern and Southern lineages of túngara frogs reside in different habitats in Panama and Costa Rica. While the Northern lineage occupies dry forests, the Southern lineage occurs in moist forests with higher annual precipitation. The actual distribution of northern and southern túngara frogs in Middle America [44] generally corresponds to the predicted suitable habitat of niche models in Costa Rica and Panama. The low predicted suitability for the Southern lineage across the distribution gap in Costa Rica is consistent with the known species distribution. Suitability across the gap for the Northern lineage, however, was intermediate and within the range of known localities. Thus, the model suggests that conditions in a portion of the gap might be suitable for the Northern lineage. This incorrect prediction of the model could be the result of (1) the exclusion of an environmental variable that limits the species distribution range, (2) ecological interactions with other species across the gap (e.g., competitive exclusion or predation), or (3) an unknown dispersal barrier. Because there are not conspicuous dispersal barriers in the region, we suspect that explanations (1) or (2) are the most likely. Niche modeling suggests that the absence of each lineage in the distribution gap correlates with ecological variables and therefore suggests that not only do the two lineages inhabit different habitats but there might be ecological divergence between the lineages. Niche conservatism is the tendency of species to retain aspects of their fundamental niche over longer time periods and limits the adaptation to ecological conditions at geographic barriers [45]. Our results show that the Northern and Southern lineages occupy different environmental envelopes and this suggests that their niches have been evolutionarily labile. There are two possibilities to explain genetic and ecological divergence in túngara frogs. The first possibility is that túngara frogs were split in two groups by a vicariance event. The geographic separation was then maintained by niche conservatism and followed by niche specialization. Otherwise the genetic divergence between lineages in túngara frogs in allopatry could be the result from a combination of niche specialization followed by niche conservatism at the edges of distribution ranges in Costa Rica. The absence of the Southern lineage in the gap suggests that niche conservatism can promote allopatric speciation as proposed by Wiens and Graham (2005) [45]. At the moment our data cannot discriminate between both possibilities for lineage separation.

In comparison with the present, suitable habitat during the last glacial maximum covered larger areas for the Northern lineage while it was more restricted in the Southern lineage. We assume that climatic changes from colder and drier climate to warmer and moister climate are responsible for this development. Drier climate during the last glacial maximum also seems to explain the higher habitat resistance of the distribution gap for the Southern lineage. Since habitat resistance persists today it seems improbable that both groups will come into contact in the near future unless the current climate change will result in more favorable conditions across the gap.

### Speciation in túngara frogs

This and several preceding molecular studies revealed that the túngara frog *Physalaemus pustulosus *consists of two genetically different lineages [34,35,39]. In this study we found that the lineages inhabit different habitats and thus suggests the possibility that they might be ecologically divergent. The geographic separation and the large genetic divergence and ecological dissimilarity between lineages suggest vicariance events together with ecologically mediated divergent selection as potential speciation mechanisms in túngara frogs. For Ecuadorian frogs of the genus *Epipedobates*, sister species with allopatric or parapatric distribution replace each other altitudinally or latitudinally emphasizing the role of environmental niche shift in speciation in neotropical frogs [21]. The ecological separation principally occurs along a temperature/elevational or a seasonality axis. For divergence between túngara frog lineages a shift in seasonality and precipitation might be more important than differences in temperature. Ecological prezygotic isolation could arise as a result of adaptation to contrasting habitats or temporal isolation [46]. Adaptation to different habitats could entail behavioral reproductive isolation because signals involved in mate choice could be under ecologically based divergent selection. Examples include sexual preferences for ecologically selected body size and color pattern in fish or beak size in birds [20]. This possibility needs further exploration in our study species.

Ron et al. (2006) [39] suggested that each lineage of túngara frogs might deserve species status based on the large molecular divergence (comparable to the divergence observed between closely related, uncontroversial species pairs), size differences, and large divergence time (6-10 My, estimates in [35]). The observed habitat differences are consistent with the recognition of each lineage as a separate species. Species level divergence is also suggested by some evidence of prezygotic isolation. Pröhl et al. (2006) [34] conducted experiments showing that captive *P. pustulosus *females from the South did not produce nests with males from the Northern lineage. However this was not true for northern females which produced nests and tadpoles with males from both lineages. The pattern of call divergence in the gap region in Costa Rica shows that several call variables tend to be more divergent near the gap. This pattern is consistent with ancient reproductive character displacement (Figure 3 in Ref. [34]), however, as discussed below, these differences in calls have no influence on female species recognition.

The results of phonotactic mate choice experiments with female túngara frogs, on the other hand, do not suggest the two lineages should each be given species status. Pröhl et al. (2006) [34] showed that calls differ between lineages and populations, and that in about two thirds of the comparisons females showed a significant preference for the local mating call over the foreign call. This preference for the local call was stronger than that found in females from Gamboa, which is well within the southern group [47]. Neither study, however, suggested that females exhibited stronger preferences for calls of their own lineage versus the other lineage (*P *= 0.32, [34]; *P *= 0.80, [47]). At this point we feel that the data are not conclusive in determining whether the two lineages of túngara frogs represent distinct species and we propose to collect more data on pre- or post-zygotic reproductive isolation [48].

### Population genetics, ecology and behavior

The results of this study offer the opportunity for new insights on the complex relationship between genetic distance, ecological divergence, call divergence and mate preferences. The areas between two genetic population clusters and areas adjacent to admixture zones are especially interesting for further behavioral observations. Such studies might determine whether there are abrupt changes in certain call parameters across genetically different populations, or how the female preference pattern varies across admixture zones. Therefore it would be necessary to conduct geographically fine scaled genetic and behavioral analyses in several localities, for example between Palmar Norte and Caracol in South Costa Rica or between Galique and El Forastero in Western Panama (Figure 1). Also, in the context of reproductive character displacement and reinforcement [49,50] coupled with measurement of gene flow, such areas provide exciting possibilities for future research prospects. The pattern of call divergence in the gap region in Costa Rica mentioned above indicates the opportunity for the evolution of character displacement in túngara frogs. Moreover the detected differences in ecology between the two main lineages should influence behavior. The northern frogs inhabit drier forests than the southern frogs. We would assume that temperature and precipitation pattern influence the time of the breeding period and the reproductive behavior. In addition the question whether there is ecological divergence and if it has caused reproductive isolation needs to be examined. Finally more intensive population sampling in the southern genetic clusters could reveal ecological differences between them and help to discover local adaptations that might be involved in divergence between lineages.

## Conclusions

In this study we found population genetic structure and unexpected ecological diversity between lineages of a Neotropical frog species, the túngara frog *Physalaemus pustulosus*. Population genetic analyses of Middle American populations (Costa Rica and Panama) revealed two allopatric divergent lineages (North and South) and a further sub-division of the Southern lineage into three population clusters. The genetic divergence into two main lineages goes along with ecological differences in habitat type. These results highlight the importance of intensive population sampling across large ranges of a species distribution. Overall genetic diversity within populations is high across the sampled range but diversity is higher in the South than in the North. The diversity within populations is maintained by high gene flow between neighbor sample localities. Altogether the genetic data support the idea that the túngara frog is an abundant species whose populations within population clusters are well connected via gene flow and little affected by habitat fragmentation. The allopatric distribution north and south of the gap in Costa Rica seems to be maintained by unsuitable climatic conditions across the gap which likely persisted during the last glaciation and probably during previous glacial and interglacial cycles. Since the Northern and Southern lineages are geographically separated, genetically divergent, and inhabit different habitats there is potential for speciation by ecological divergence in allopatry. We discuss the possibility that both lineages should receive species status and conclude that additional pre- and postzygotic isolation experiments are needed. In addition, further ecological and behavioral analyses among and across southern population clusters could shed light on evolutionary divergence and speciation.

## Methods

### Studied species and population sampling

The studied species is the Leiuperid frog, *Physalaemus pustulosus*. It occurs in wet and dry regions, and can be abundant in disturbed and undisturbed habitats. During the last few years there has been controversy on its generic assignment under either *Physalaemus *or *Engystomops*. Because neither alternative creates paraphyly [51,52] the choice between alternatives is a matter of preference between nomenclatural stability (favored by MJR) and nomenclatural informativeness (favored by SRR).

Field collection, DNA extraction, the genetic analysis and genotyping of microsatellites were described in detail in [34]. To summarize, from July to October 2000 we collected tissues from 18 Costa Rican and 6 Panamanian túngara frog populations from Santa Rosa National Park in the northwest (Costa Rica) to Santiago in the southeast (Panama), a straight line distance of 565 km (Figure 1). In addition we collected tissues from Gamboa in central Panama which is 188 km northeast from Santiago. The sampled area included eight population in the Northern lineage and 17 populations in the Southern lineage as defined by Ryan et al. (1996) [32] and Pröhl et al. (2006) [34]. We removed one toe tip from an average of 19 frogs per population and stored the tissue in NaCl-saturated 20% DMSO/0.25M EDTA buffer at room temperature. We also documented longitude and latitude of each sample and calculated geographic distances between sample sites using a 12 channel GPS (Garmin, Taipei, Taiwan).

### Laboratory Methodology

#### Microsatellites

We extracted DNA with the DNeasy Tissue Kit (Qiagen, Valencia, CA) and subsequently amplified six highly polymorphic microsatellites loci (C30.11, ATG159, CA298, A3.11, A19.11, ATG263) for all sampled frogs (N = 457) with primers and respective amplification methods for PCR previously developed for túngara frogs [53]. PCR products were analyzed on the ABI (Applied Biosystems, Foster City, CA) 3100 Genetic Analyzer and then scored using Gene Scan Analysis Version 3.5 (ABI) and Genotyper version 3.6 NT (ABI) software. We include all six loci in our analysis because we did not uncover linkage disequilibrium for any loci pair in any population and observed heterozygosity deviated from expected heterozygosity in only one locus in few populations [34]. In total, 199 alleles were found at the six loci, ranging from one to eight (ATG 263) to as many as six to 20 alleles (A19.11) in a single population. Although the number of microsatellite loci is relatively small, they are highly polymorphic and the large number of alleles allows substantial resolution at the population level as simulation studies have shown that the number of independent alleles, rather than the number of loci, is critical for estimating genetic distances [54]).

#### Cytochrome B

To verify population structure based on microsatellite we also sequenced mitochondrial *Cyt B *of a limited number of individuals. Sequences were obtained from one to two individuals per sample locality (total N = 37). A 487-base-pair (bp) segment was amplified using the primers MVZ15-L (5'-GGACTAATGGC CCAC ACWWTACGNAA-3'; [55]) and CytbAR-H (5'-TAWAAGG GTCTTCTACTAC TGGTTG-3'; [56]). Polymerase chain reaction (PCR) amplifications were carried out in a total volume of 25 μL using approximately 10 ng of frog DNA, 0.8 mM of each dNTP, 2.5 μL 10 X advanced PCR Buffer containing self-adjusting MgCl2 (Eppendorf Deutschland, Hamburg), 1.25 U Taq-Polymerase (THH Pyrophosphatase, Invitek, Berlin) and 10 pmol of both forward and reverse primers. Amplification was performed in the Eppendorf Mastercycler ep*gradient *(Eppendorf Deutschland, Hamburg) under the following conditions: 94°C for 3 min, followed by 35 cycles of 94°C for 45 s, 50°C for 45 s, 65°C for 60 sec. PCR products were sequenced in both directions by the Macrogen Sequencing Team (Macrogen Inc., Seoul, Korea). *Cyt B *sequences were assembled and edited using the SeqMan module of the Lasergene program (DNASTAR Inc., Madison, Wis.) and aligned in Clustal X version 1.8 [57] using default settings. Sequences were deposited in GenBank (accession numbers GU086726-GU086762) [Additional file 5].

### Statistical analysis

#### Microsatellites

Population structure: A fundamental requirement of any inference about the genetic structure of populations is the definition of populations themselves. Pritchard et al. (2000) [58] developed a method which is implemented in the software Structure. This method aims to define clusters of individuals on the basis of their genotypes at multiple loci using a Bayesian approach. The method attempts to find population clusters by minimizing linkage disequilibrium and deviations from the Hardy-Weinberg equilibrium within inferred clusters. The user defines the number of population clusters (K) and estimates the log likelihood of the hypothesis given by each value of K: Pr (X|K) [Estimated Ln Prob of data = *Ln P I D *gives the value for Pr (X|K) in the software result output].

The authors of the Structure software point out that it is not always possible to know the true value of K; they recommend that one may aim for the smallest value of K that captures the major genetic structure in the data. This can be achieved by selecting the lowest K when several values of K give similar estimates of Pr (X|K). For example in case that Pr (X|K) plateaus for higher values of K one would choose the K instead of K+1 when the difference in Pr (X|K) to Pr (X|K + 1) is small.

The problem to detect the true number of clusters was addressed by Evanno et al. (2005) [59]. They found that in many cases Pr (X|K) does not provide a correct estimation of the number of clusters, K. They propose to use the statistic delta K (= ΔK) which relates to the second order rate of change of the log likelihood of the data Pr (X|K) with respect to K. By testing different scenarios (different types of genetic markers, different number of loci, different number of individuals and populations scored) they found that ΔK is a good predictor of the real number of clusters. For our study we estimated Pr (X|K) along with ΔK for K = 1 to K = 10. For the analysis we used the default values of most parameters as proposed in the user manual of Structure[60]. We applied the admixture model and the option of correlated allele frequencies between populations as recommended by Falush et al. (2003) [61] for situations with slight population structure. We selected the length of burn-in and the number of MCMC (Markov Chain Monte Carlo) replications after burn-in both to be 50,000. Since different runs can results in different Pr (X|K) values, 20 runs were carried out for each K and mean values of Pr (X|K) were calculated. To estimate genetic differentiation between the resulting population clusters of the Structure analysis we calculated F_ST _[62] and R_ST _values [63] with the software Arlequin 2.0 [64].

Migration and assignment of individuals to populations: We used the Program GENECLASS2 to assign individuals to one or more sample localities based on their allele frequencies. The method we used to assign individuals probabilistically to sample localities was the partial Bayesian method [65]. As a result, for each individual the five most likely populations of origin and their relative assignment scores are given in decreasing order. The assignment threshold of scores was 0.05. We distinguish between individuals that were assigned with the highest score to their sample locality, individuals that were assigned with the highest score to another sample locality from the same population cluster and individuals that were assigned to a sample locality from another population cluster as initially identified by Structure.

The same program was also used to detect first generation migrants (F0): We used the test statistic **Λ **= L_home/L_max, which is the ratio of the likelihood computed from the population where the individual was sampled (L_home) over the highest likelihood value among all population samples including the population where the individual was sampled (L_max; i.e. the likelihood for the population to which the individual would be assigned in the assignment test; see [66]) to detect first generation migrants (F0). We selected the frequency based method as the criterion for the likelihood computation [66]. We calculated the probability that an individual is a resident by running a Monte Carlo resampling algorithm recommended for first generation migrant detection [66]. The number of simulated individuals was 1000 while the type I error (a) was set at 0.01.

For comparison we also applied the classical method from Wright (1943) [5] for indirectly estimating gene flow among population by means of F_ST _and R_ST _values. The number of migrants per generation is estimated as follows: Nm = (1-F_ST_)/(4 F_ST_). Although some assumptions of population structure (infinite island model of population structure and gene flow) might be violated in most real situations this estimate is still considered a useful instrument for comparative purposes (e.g. [67]). The generation duration in túngara frogs is approximately one year [68].

Genetic diversity: We used the Program FSTAT to calculate allelic richness as a measure of genetic diversity of each population. Allelic richness measures the number of alleles per locus and is highly dependent on effective population size. Allelic richness must be standardized to cope with uneven samples sizes. This is achieved by applying a rarefaction technique [69] which was first introduced into ecological studies. The *OSx*-Statistic implemented in FSTAT was used to test for differences in allelic richness among population clusters. One thousand permutations were run to test the significance of the results.

Isolation by distance: To investigate the effect of gene flow over distance we used Mantel tests [70] implemented in Arlequin 2.0 [64] to examine the correlation between geographic distance and genetic distance. We included three models for analyzing this relationship: (1) straight line geographic distance versus R_ST _estimates for pairs of subpopulations; (2) natural logarithm of geographic distance versus R_ST _values; and (3) natural logarithm of geographic distance versus R_ST_/(1-R_ST_). The last two approaches have been proposed [71] for the F_ST _statistic to linearize the relationship between F_ST _and distance; (the 2nd. and 3rd. approach have been suggested for larger and smaller distances between populations, respectively). The first approach, however, yielded the best results (highest correlation coefficient and coefficient of determination) in our study and only these data are presented here. We used R_ST _instead of F_ST _because F_ST _tends to underestimate genetic differentiation when applied to microsatellite data [63] and this seems to be also true for túngara frogs [34].

#### Cytochrome B

To estimate genetic differentiation based on nucleotide differences we used MEGA 4.0 [72] to calculate uncorrected p-distances and TN distances [73]. We also present TN distance here because it presents a general case of the HKY model, and takes into account differences in substitutions rates between transitions and transversions. The HKY+G model [74] was identified in MODELTEST as the most likely substitution model by the hierarchical likelihood ratio test (hLRT) for the *Cytochrome B *(*Cyt B*) sequences. We estimated time of divergence between genetic clusters by uncorrected p-distance divided by the pairwise evolutionary rate/MYR.

We used *Cyt B *to determine the divergence time between the Northern and Southern lineage and the population clusters of túngara frogs, because this gene has been proven useful for intraspecific and genus level relationships in several amphibian species [2,75-77]. Evolutionary rates of mitochondrial genes have been found to be relatively constant across diverse poikilothermic vertebrate species [78,79]. Rates of mtDNA evolution were estimated to be 0.69% sequence divergence per million years per lineage in the *Bufo bufo *species group [78]), 0.64% in hynobiid salamanders [79]), and 0.7-0.8% in north American salamandrids [80]). Taken together these studies suggest a pairwise rate of change of approximately 1.3-1.4% sequence divergence per million years in amphibian mitochondrial genes (but see [2,81]). Similar evolutionary rates have therefore been applied for divergence times for other amphibian species [13,75,82]. Therefore for our analysis we assumed an evolutionary rate of 0.7%/MYR per lineage which is the same as 0.7 substitutions/site/100 MYR. We estimated divergence time between population clusters by only applying a standard molecular clock since Weigt et al. (2005) [35] found a reasonable correspondence in dates and rates between the "frog clock" and parametric Bayesian methods in *P. pustulosus*.

We used TCS vs. 1.21 [83] to construct a haplotype network of *Cyt B *sequences. The program calculates the frequencies of each haplotype and the number of mutational steps between two haplotypes associated with the 95% limit for the probability of parsimony ('parsimony' criterion [84]). Finally we calculated indices of molecular diversity (number of haplotypes, number of polymorphic sites, haplotype (= gene) diversity and nucleotide diversity) of the *Cyt B *sequences applying DnaSP vs. 4.50.3 [85] for the total population, for genetic lineages and for all population clusters.

### Niche Modeling

We employed environmental niche modeling to: (1) predict the current and past distribution of suitable habitat for túngara frogs, (2) to compare the connectivity across the distribution gap in Costa Rica between present and glacial conditions, and (3) to assess the level of ecological divergence between the Northern and Southern lineage. Niche models are based on environmental values at localities of known occurrence of the target taxa, which then are used to identify geographic regions that have similar combinations of values. The input for model building consists of (1) a set of localities of known occurrence of the target species, and (2) environmental data from digital maps (e.g., annual temperature, annual precipitation, altitude) for the target region. Niche models were obtained with Maxent, a maximum entropy algorithm that generates a probability distribution of habitat suitability across the target region [86]. We chose Maxent among several modeling options because of its high efficiency and predictive performance [86-89]. Maxent operates under the maximum-entropy principle, which seeks to generate a probability distribution of habitat suitability that is closest to uniform (i.e., with equal probabilities of occurrence in all map grid cells) but subject to the constraints imposed by sets of environmental values at localities of known occurrence of the species. The probability distribution assigns a habitat suitability value to each grid in the map (see for a description of its mathematical definition and its use in environmental niche modeling). We ran the analyses using Maxent version 3.2.1 [90] under the default modeling parameters: convergence threshold = 10^-5^, maximum iterations = 500, regularization multiplier = 1.0. The logistic output from Maxent is a raster map with grid cell values ranging from 0 to 1, which can be interpreted as the probability of presence of suitable habitat for the species [89].

Túngara frog localities were obtained from [34,35], fieldwork by HP and MJR and records from natural history collections: Museum of Vertebrate Zoology at the University of California Berkeley, Natural History Museum at the University of Kansas, and Royal Ontario Museum. Museum records were accessed through the HerpNET data portal [91]. Localities without coordinates were georeferenced using the gazetteers from the Alexandria Digital Library Project [92]. To reduce spatial autocorrelation, we only included localities separated by at least 10 km.

The environmental data for niche modeling consisted of 12 raster maps (11 bioclimatic variables and altitude; resolution = 10 km × 10 km per cell) obtained from WorldClim [88]). The bioclimatic rasters are: (1) annual mean temperature, (2) mean temperature diurnal range, (3) isothermality, (4) temperature seasonality, (5) maximum temperature of warmest month, (6) mean temperature of driest quarter, (7) mean temperature of coldest quarter, (8) annual precipitation, (9) precipitation of wettest month, (10) precipitation seasonality, and (11) precipitation of driest quarter.

To estimate the past distribution of suitable habitat for túngara frogs, we projected the environmental niche model to climate conditions for the last glacial maximum (LGM; ~21,000 BP). The LGM climate raster maps are based on the palaeoclimate ECHAM3 model and were assembled as described by Hijmans and Graham (2006) [88] and Ruegg et al. (2006) [93].

Niche models were built independently for the Northern and the Southern lineages. Models were based on all available localities (i.e. 9 in western Costa Rica for the northern group; 21 in eastern Costa Rica and Panama for the southern group) except in tests of model performance. To tests model performance, the southern localities were partitioned into two halves with random assignment: a training and a testing set. The training set was used for model building and the testing set for model evaluation. By setting an arbitrary threshold, the logistic output of the model was transformed into a binary map (suitable vs. unsuitable conditions). Then, we applied a binomial test to compare the proportion of localities from the testing set correctly predicted within the suitable habitat vs. the expected proportion for a random model with the same amount of suitable habitat (e.g., a random model with suitable habitat on 50% of the total area is expected to include 50% of the localities just by chance). This procedure was repeated 10 times, each for a random partition of localities. In each replicate, the binomial test was applied to 10 commonly used logistic thresholds. Because the northern group was built with only 9 localities, we used as testing set the localities of known presence of túngara frogs outside the study region (Nicaragua to Mexico). Model performance was also evaluated with the receiver operating characteristic (ROC) analysis, which consists of a sensitivity analysis of the presence and absence points predicted by the model [86].

Connectivity and suitability across the distribution gap in Costa Rica: The two most divergent genetic lineages (North-South) are separated by a ~200 km distribution gap in Costa Rica. This suggests that genetic divergence is a result of geographic isolation. Because distribution ranges change with changing environmental conditions, we do not know whether both ranges were also unconnected during glacial times. To explore this question, we developed a new measure of landscape connectivity based on ecological niche modeling. Landscape connectivity is the degree to which the landscape allows movement across the distribution gap. We assumed that the resistance to movement across habitat is an inverse function of habitat suitability, as defined by the niche models. We obtained a raster map of habitat resistance by subtracting one minus the niche suitability values from the logistic output (ranging from 0 to 1). Based on this raster, we estimated the least-cost distance path [94] between the distribution borders of both genetic lineages in Costa Rica. A high value of this metric will indicate that habitat resistance to movement is high and thus genetic flux more difficult. If the analyses show that the gap currently separating both ranges was more resistant to dispersal during the last glaciation, we will have much greater confidence in the role of geographic isolation as the main cause for genetic divergence. The least-cost distance path was calculated with the Connectivity Tool (version 2) for ESRI ArcGIS(r) [95]. To test for differences in habitat suitability between the last glacial maximum and current conditions on the distribution gap, we compared, with a Student's paired *t*-test, the LVs for the present model vs. the LVs for the glacial model contrasting overlapping pixels in the gap (hence the paired test).

## Authors' contributions

HP designed the study, collected samples, carried out the molecular studies, performed statistical analyses, and drafted the manuscript. SRR performed niche-modeling analysis, interpreted data and helped writing the manuscript. MJR participated in the design of the study, interpreted data and helped draft the manuscript. All authors read and approved the manuscript.

## Supplementary Material

Additional file 1 **Pairwise R_ST _values among Middle American populations of túngara frogs (*Physalaemus pustulosus*)**. Matrix of pairwise R_ST _values based on microsatellites between all sampled populations of túngara frogs. Click here for file

Additional file 2 **Assignment of individuals to sample localities**. All individuals were genetically assigned to sample localities by the program GENECLASS2. The figures present the % of individuals in every sampled (= "home") population for those the most likely "source" population is the "home" population (black bars), the most likely "source" population is another population from the same population cluster (grey bars), the most likely "source" population is from another population cluster (light grey bars); A) Population cluster North, B) Population cluster South_1, C) Population cluster South_2, D) Population cluster South_3. Click here for file

Additional file 3 **First generation migrants**. List of first generation migrants detected by GENECLASS2: the table contains all individual frogs which probably migrated from a "source" population to their "home" population (= sample locality). Click here for file

Additional file 4 **Genetic diversity in population clusters**. Genetic diversity as measured by allelic richness with FSTAT in all populations organized by population clusters. Click here for file

Additional file 5 **GenBank accession numbers for *Cyt B *sequences**. Sampling localities, genetic clusters and GenBank accession numbers of all *Cyt B *sequences from túngara frogs analyzed in this study. Click here for file
