# Supplementary material for: Ecological and genetic divergence between two lineages of Middle American túngara frogs Physalaemus (= Engystomops) pustulosus
Source: BMC Evol Biol. 2010 May 18;10:146. doi: 10.1186/1471-2148-10-146 (PMC2882927; doi:10.1186/1471-2148-10-146)
Supplement: Additional file 2 — Assignment of individuals to sample localities. All individuals were genetically assigned to sample localities by the program GENECLASS2. The figures present the % of individuals in every sampled (= "home") population for those the most likely "source" population is the "home" population (black bars), the most likely "source" population is another population from the same population cluster (grey bars), the most likely "source" population is from another population cluster (light grey bars); A) Population cluster North, B) Population cluster South_1, C) Population cluster South_2, D) Population cluster South_3. [file 1471-2148-10-146-S2.PDF]

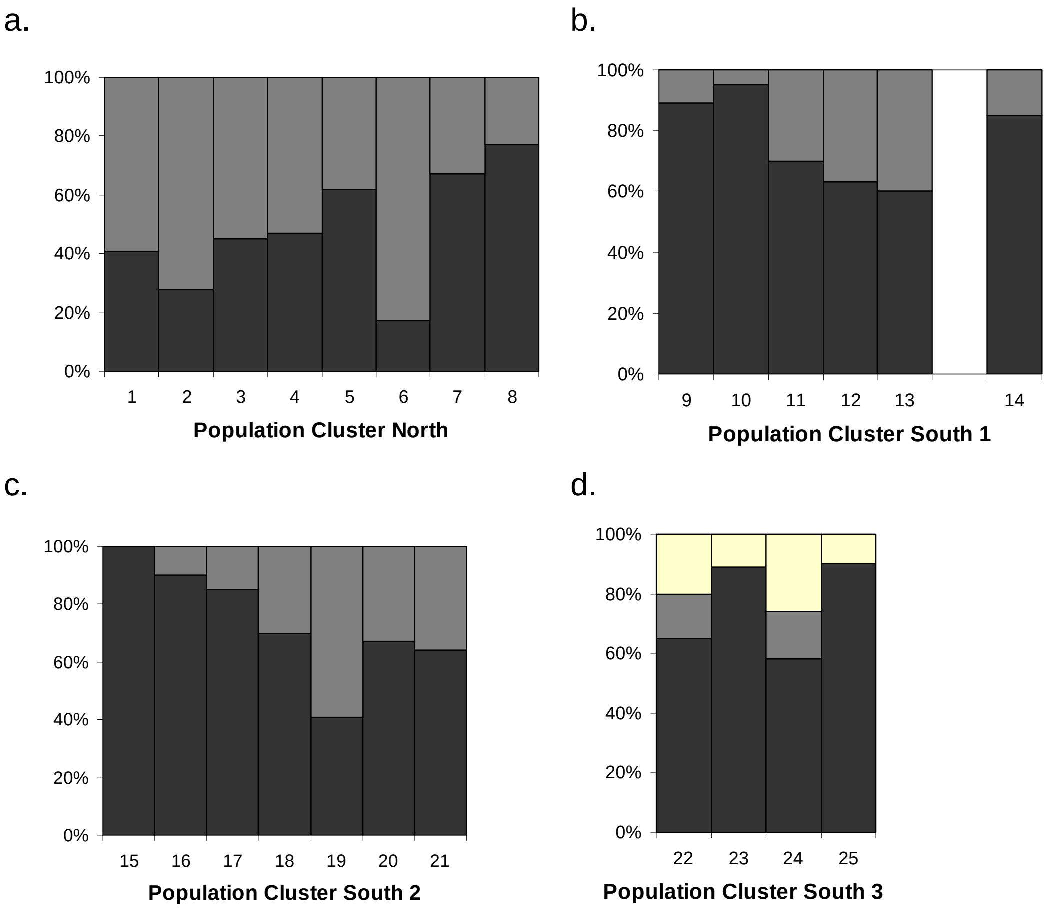

Supplemental Figure S1 - Assignment of individuals to sample localities
Percent of individuals in every home population for those the most likely source population is the home population (black bars), the most likely source population is another population from the same population cluster (grey bars), the most likely source population is from another population cluster (light grey bars). A: for the population cluster North; B: for the population cluster South_1 and admixture zone (14 = Piedras Blancas); C: population cluster South_2; D: population cluster South_3. The sample localities are indicated by numbers: 1=Santa Rosa, 2=Liberia, 3=Agua Caliente, 4=Filadelfia, 5=Nicoya, 6=Palma, 7=Peñas Blancas, 8=La Junta, 9=Ochojal, 10= Buenos Aires, 11= Cortéz, 12=Potrero Grande, 13=Palmar Norte 14=Piedras Blancas, 15=Osa, 16=Golfito, 17=Caracol, 18=Gloria, 19=Gariché, 20=Bugaba, 21=El Forastero, 22=Galique, 23=Tolé, 24=Santiago, 25=Gamboa.
