# Supplementary material for: Ecological and genetic divergence between two lineages of Middle American túngara frogs Physalaemus (= Engystomops) pustulosus
Source: BMC Evol Biol. 2010 May 18;10:146. doi: 10.1186/1471-2148-10-146 (PMC2882927; doi:10.1186/1471-2148-10-146)
Supplement: Additional file 3 — First generation migrants. List of first generation migrants detected by GENECLASS2: the table contains all individual frogs which probably migrated from a "source" population to their "home" population (= sample locality). [file 1471-2148-10-146-S3.PDF]

Supplemental Table S2 – First generation migrants

Detection of first generation migrants: individuals which are probably emigrated from another sample locality.

Cluster	Home Population / Individual	-log(L-home/L-max)	Probability that the individual is a resident	Most likely source population	Distance (km) between home and source population
North	Santa Rosa /17	2.37	0.0095	Liberia	32
	Filadelfia / 2	2.69	0.0009	Liberia	21
	Nicoya / 13	2.09	0.0097	Liberia	53
	Peñas Blancas / 4	2.64	0.0016	La Junta	19
	La Junta / 4	1.98	0.0049	Liberia	65
	21	1.60	0.0071	Palma	9
South 1	Ojochal / 9	0.69	0.0053	Cortez	20
	Buenos Aires / 6	0.75	0.0069	Cortez	30
	Cortez / 24	3.08	0.0011	Potrero Gr.	39
	35	2.57	0.0089	Palma Norte	7
	Potrero Gr. / 7	2.67	0.0015	Palma Norte	32
Admixture zone	Piedras Bl. / 1	1.991	0.0069	Bugaba	75
South 2	Caracol / 5	2.35	0.0048	Gloria	21
	Gloria / 4	2.51	0.0041	Gariche	11
	El Forastero / 5	3.44	0.0068	Osa	116
	18	2.92	0.0047	Bugaba	21
South 3	Galique / 6	2.16	0.006	Tolé	29

Tole / 2	2.06	0.0032	Piedras Bl.	184
15	1.24	0.0072	Santiago	54
16	2.31	0.0035	Golfito	174
Santiago / 10	2.11	0.0019	Caracol	206
18	1.72	0.0038	Tole	54
Gamboa / 3	2.19	0.0015	Cortez	457
4	0.23	0.0081	Caracol	395
