# Supplementary material for: Ecological and genetic divergence between two lineages of Middle American túngara frogs Physalaemus (= Engystomops) pustulosus
Source: BMC Evol Biol. 2010 May 18;10:146. doi: 10.1186/1471-2148-10-146 (PMC2882927; doi:10.1186/1471-2148-10-146)
Supplement: Additional file 4 — Genetic diversity in population clusters. Genetic diversity as measured by allelic richness with FSTAT in all populations organized by population clusters. [file 1471-2148-10-146-S4.PDF]

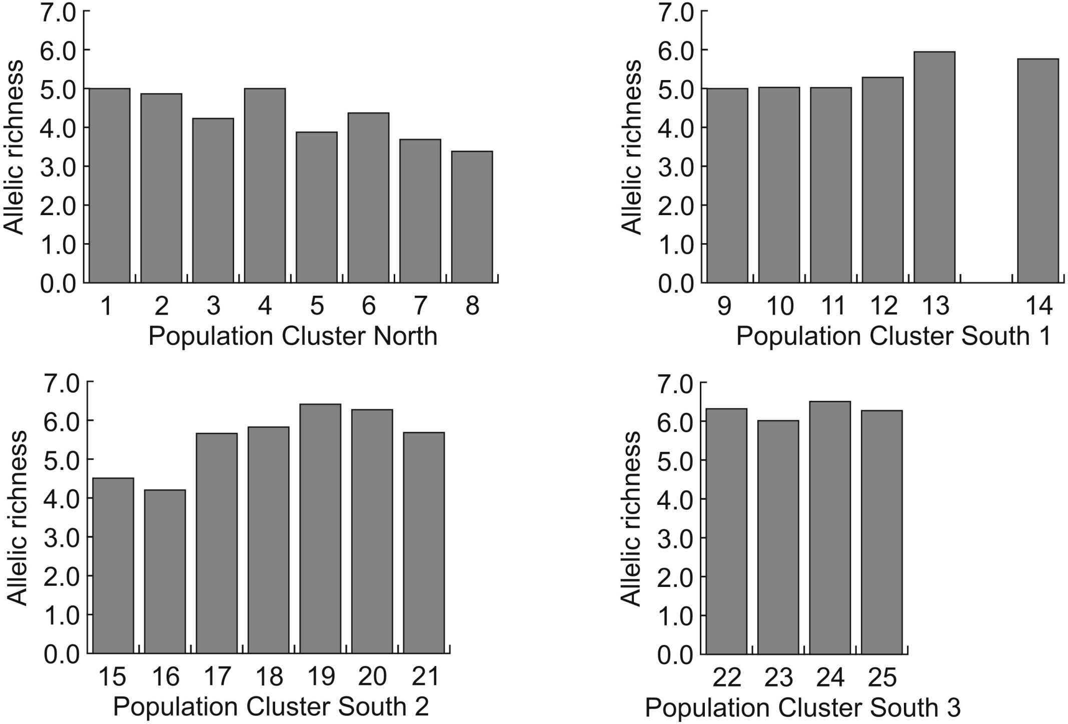

Supplemental Figure S2 - Genetic diversity in population clusters
Genetic diversity as measured by allelic richness in all populations organized by pobulation clusters. Populations: see legend of Additional File 2.
