# Supplementary material for: Ecological and genetic divergence between two lineages of Middle American túngara frogs Physalaemus (= Engystomops) pustulosus
Source: BMC Evol Biol. 2010 May 18;10:146. doi: 10.1186/1471-2148-10-146 (PMC2882927; doi:10.1186/1471-2148-10-146)
Supplement: Additional file 5 — GenBank accession numbers for Cyt B sequences. Sampling localities, genetic clusters and GenBank accession numbers of all Cyt B sequences from túngara frogs analyzed in this study. [file 1471-2148-10-146-S5.PDF]

Supplemental Table S3 – GenBank accession numbers for *Cyt B* sequences

Sampling localities of túngara frogs, *Physalaemus pustulosus,* assignment to genetic cluster, number of individuals sequenced for Cyt B and GenBank accession numbers.

	Sampling locality	Genetic Cluster	Cyt B	GenBank accession number
1	Santa Rosa	North	1	GU086759
2	Liberia	North	1	GU086746
3	Agua Caliente	North	1	GU086726
4	Filadelfia	North	2	GU086734
				GU086735
5	Nicoya	North	1	GU086747
6	Palma	North	1	GU086751
7	Peñas Blancas	North	1	GU086754
8	La Junta	North	2	GU086744
				GU086745
9	Ochojal	South_1	2	GU086748
				GU086749
10	Buenos Aires	South_1	2	GU086727
				GU086728
11	Cortéz	South_1	1	GU086731
12	Potrero Grande	South_1	2	GU086757
				GU086758
13	Palmar Norte	South_1	2	GU086752
				GU086753
14	Piedras Blancas	Admixture Zone	2	GU086755
				GU086756
15	Osa	South_2	1	GU086750
16	Golfito	South_2	1	GU086743
17	Caracol	South_2	1	GU086730
18	Gloria	South_2	1	GU086742
19	Gariché	South_2	2	GU086740
				GU086741
20	Bugaba	South_2	1	GU086729
21	El Forastero	South_2	2	GU086732
				GU086733
22	Galique	South_3	1	GU086736
				GU086737
23	Tolé	South_3	2	GU086761
				GU086762
24	Santiago	South_3	1	GU086760
25	Gamboa	South_3	2	GU086738
				GU086739
